# Supplementary material for: Geographic spillover of antimicrobial resistance from mass distribution of azithromycin
Source: Nat Commun. 2026 Jan 29;17:2152. doi: 10.1038/s41467-026-68691-y (PMC12957345; doi:10.1038/s41467-026-68691-y)
Supplement: Supplementary file 2 — Reporting Summary [file 41467_2026_68691_MOESM2_ESM.pdf]

nature portfolio

Corresponding author(s): Benjamin F. Arnold

Last updated by author(s): Dec 22, 2025

Reporting Summary

Nature Portfolio wishes to improve the reproducibility of the work that we publish. This form provides structure for consistency and transparency in reporting. For further information on Nature Portfolio policies, see our Editorial Policies and the Editorial Policy Checklist.

Statistics

For all statistical analyses, confirm that the following items are present in the figure legend, table legend, main text, or Methods section.

n/a	Confirmed	
☐	☒	The exact sample size (*n*) for each experimental group/condition, given as a discrete number and unit of measurement
☐	☒	A statement on whether measurements were taken from distinct samples or whether the same sample was measured repeatedly
☐	☒	The statistical test(s) used AND whether they are one- or two-sided<br>*Only common tests should be described solely by name; describe more complex techniques in the Methods section.*
☐	☒	A description of all covariates tested
☐	☒	A description of any assumptions or corrections, such as tests of normality and adjustment for multiple comparisons
☐	☒	A full description of the statistical parameters including central tendency (e.g. means) or other basic estimates (e.g. regression coefficient) AND variation (e.g. standard deviation) or associated estimates of uncertainty (e.g. confidence intervals)
☐	☒	For null hypothesis testing, the test statistic (e.g. *F*, *t*, *r*) with confidence intervals, effect sizes, degrees of freedom and *P* value noted<br>*Give P values as exact values whenever suitable.*
☒	☐	For Bayesian analysis, information on the choice of priors and Markov chain Monte Carlo settings
☐	☒	For hierarchical and complex designs, identification of the appropriate level for tests and full reporting of outcomes
☐	☒	Estimates of effect sizes (e.g. Cohen's *d*, Pearson's *r*), indicating how they were calculated

Our web collection on statistics for biologists contains articles on many of the points above.

Software and code

Policy information about availability of computer code

Data collection	There was no data collection software used in this secondary analysis of data from 2 cluster randomized trials.
Data analysis	All code is available: https://osf.io/qxtec/. Analyses used R statistical software (version 4.4.0, 2024-04-24 "Puppy Cup"). Key packages used are: broom 1.0.11, cowplot 1.2.0, ggpubr 0.6.2, ggspatial 1.1.10, ggtext 0.1.2, here 1.0.2, lwgeom 0.2-14, patchwork 1.3.2, raster 3.6-32, sf 1.0-23, tidyverse 2.0.0, units 1.0-0. Full list available at: https://osf.io/x4e3n.

For manuscripts utilizing custom algorithms or software that are central to the research but not yet described in published literature, software must be made available to editors and reviewers. We strongly encourage code deposition in a community repository (e.g. GitHub). See the Nature Portfolio guidelines for submitting code & software for further information.

Data

Policy information about availability of data

All manuscripts must include a data availability statement. This statement should provide the following information, where applicable:

- Accession codes, unique identifiers, or web links for publicly available datasets
- A description of any restrictions on data availability
- For clinical datasets or third party data, please ensure that the statement adheres to our policy

The trial data used in this analysis have been deposited in Open Science Framework (https://doi.org/10.17605/OSF.IO/BMJD3). Village geographic coordinates data are protected and are not available to protect participant confidentiality. The microbial sequencing reads have been deposited with the NCBI Sequence Read Archive under BioProject no. PRJNA1356862. Administrative boundaries used in maps were sourced from United Nations Office for the Coordination of

Humanitarian Affairs' Common Operational Datasets hosted on Humanitarian Data Exchange (accessed at https://data.humdata.org/dataset/cod-ab-ner). Population estimates were obtained from High Resolution Settlement Layer by Meta's Data for Good (accessed at https://data.humdata.org/dataset/highresolutionpopulationdensitymaps-ner). Source data are provided with this paper. Replication code for all analyses is also available through the Open Science Framework (https://osf.io/rc9qj/files/github).

Research involving human participants, their data, or biological material

Policy information about studies with human participants or human data. See also policy information about sex, gender (identity/presentation), and sexual orientation and race, ethnicity and racism.

Reporting on sex and gender	Mass azithromycin distribution was implemented at the community level to all eligible children aged 1-59 months regardless of sex, with approximately 50.7% male participants in the azithromycin group and 50.6% in the placebo group at baseline. Sex was determined based on biological attributes recorded during census registration. This secondary analysis included both male and female children. The analysis did not stratify results by sex or gender, as the spillover effects of antimicrobial resistance from mass drug administration were expected to operate at the community level rather than varying by individual-level sex or gender characteristics.
Reporting on race, ethnicity, or other socially relevant groupings	The study population consisted of children from rural Nigerien communities, representing the local ethnic and cultural groups in this region of sub-Saharan Africa. Race, ethnicity , or other social groupings were not used as analytical variables in this secondary analysis, as the focus was on geographic spillover effects of antimicrobial resistance between villages rather than differences between sociodemographic groups at an individual level.
Population characteristics	See below
Recruitment	Eligible villages were identified through a complete census of non-urban villages with estimated populations of 200-2,000 people in the study region. All villages meeting geographic and population criteria were eligible for inclusion. From this sampling frame, 594 villages were randomly selected and enrolled in the mortality monitoring trial, and an additional 30 villages were randomly selected for AMR monitoring. Within enrolled villages, all children aged 1-59 months identified during house-to-house censuses were eligible to participate and offered enrollment, reducing self-selection bias at the individual level. For the AMR monitoring component, 10 children per village were randomly selected for specimen collection at each timepoint. The primary limitation to generalizability relates to the exclusion of urban areas and villages outside the specified population range, which was appropriate for the study's focus on rural, high-mortality settings in sub-Saharan Africa.
Ethics oversight	The trial protocol was reviewed and approved by the Committee for Human Research at the University of California, San Francisco (protocol #10-01036) and the Ethical Committee of the Niger Ministry of Public Health. The trial was monitored by an independent Data and Safety Monitoring Committee.

Note that full information on the approval of the study protocol must also be provided in the manuscript.

Field-specific reporting

Please select the one below that is the best fit for your research. If you are not sure, read the appropriate sections before making your selection.

☐ Life sciences ☒ Behavioural & social sciences ☐ Ecological, evolutionary & environmental sciences

For a reference copy of the document with all sections, see nature.com/documents/nr-reporting-summary-flat.pdf

Behavioural & social sciences study design

All studies must disclose on these points even when the disclosure is negative.

Study description	This is a pre-specified secondary, quantitative analysis of the MORDOR (Macrolides Oraux pour Réduire les Décès avec un Oeil sur la Résistance) cluster-randomized, placebo-controlled trial (NCT02047981) and its sister MORDOR Morbidity trial (NCT02048007). The secondary analysis investigates between-village geographic spillover effects of antimicrobial resistance (AMR) following mass drug administration (MDA) of azithromycin in Niger's Dosso region.
Research sample	The trial was conducted in the Boboye, Loga and Falmey departments in Dosso region of Niger. All non-urban villages with an estimated population of 200-2000 people in the study area were eligible to be included in the trial. 594 villages enrolled in mortality monitoring (303 azithromycin, 291 placebo). 30 additional villages separately randomized for AMR monitoring (15 azithromycin, 15 placebo). All children aged 1-59 months residing in trial villages were eligible to receive the assigned treatment to monitor the impact of azithromycin MDA on childhood mortality. This sample is representative of rural, high-mortality settings in sub-Saharan Africa, where azithromycin MDA is being considered to reduce childhood mortality.
Sampling strategy	The mortality-monitoring MORDOR trial was powered to detect a 10% reduction in mortality with 620 communities per country, based on mortality rates of 14-20 deaths per 1,000 person-years in placebo group, average community sizes of 600-799 people (16.7-19.0% children aged 1-59 months), coefficients of variation between 0.40-0.51 and 10% loss to follow-up. The AMR monitoring substudy (15 villages per arm, 10 children per village) was designed to provide 80% power to detect an 18% difference in resistance prevalence (12% to 30%), assuming 12% baseline resistance, an intracluster correlation of 0.051, and 80% pneumococcal carriage. For the present spillover analysis, no formal sample size calculation was performed. The analysis leveraged the existing trial infrastructure with 30 AMR monitoring villages (15 per arm), each with 10 children sampled.
Data collection	Treatment administration was documented via a custom mobile application on tablet computers, with village-level coverage

	calculated relative to census data. House-to-house censuses were performed at five 6-month intervals, documenting all children aged 1-59 months, in the presence of a parent/guardian, and recording household GPS coordinates. Nasopharyngeal and rectal swabs were collected from a random sample of 10 children aged 1 to 59 months in each of the 30 AMR monitoring villages. At each visit, a registration worker assigned each child a random identification number linked to a QR code, which was placed on a colored bracelet and used to track all specimens while maintaining participant anonymity. Census workers, specimen collection teams and laboratory personnel remained masked to treatment assignments.<br><br>Geographic variables including population density and distance to health facilities were obtained from external data sources.
Timing	The study timeline spanned 24 months from December 2014 through August 2017. Baseline census and AMR sample collection occurred December 2014 - July 2015. Follow-up censuses occurred at approximately 6-month intervals, with MDA rounds following each census. Final AMR sample collection occurred February 2017 - August 2017, approximately 24 months after baseline and after the fourth round of mass drug administration.
Data exclusions	A total of 636 communities were excluded before randomization due to population outside the 200-2,000 range or not being needed for target sample size. An additional 91 villages were enrolled in parallel trials with additional data collection. In Niger, one community declined participation and 20 were excluded post-randomization due to census inaccuracies including duplicate communities or non-existent communities.
Non-participation	Mean treatment coverage was 90.3±10.6% for azithromycin and 90.4±10.1% for placebo across the four distributions, with the primary reason for non-receipt being that the child was away from the household during the distribution visit. Independent validation census of a random subset of at least 200 households showed 95% concordance with the original census. For AMR specimen collection, rectal swabs were successfully collected from 10 children per village (300 total) at both baseline and 24 months with no refusals. Streptococcus pneumoniae was isolated from 27 of 30 villages at 24 months.
Randomization	Eligible villages were randomized 1:1 to receive mass drug administration with azithromycin or placebo.

Reporting for specific materials, systems and methods

We require information from authors about some types of materials, experimental systems and methods used in many studies. Here, indicate whether each material, system or method listed is relevant to your study. If you are not sure if a list item applies to your research, read the appropriate section before selecting a response.

Materials & experimental systems

n/a	Involved in the study
☒	☐ Antibodies
☒	☐ Eukaryotic cell lines
☒	☐ Palaeontology and archaeology
☒	☐ Animals and other organisms
☒	☐ Clinical data
☒	☐ Dual use research of concern
☒	☐ Plants

Methods

n/a	Involved in the study
☒	☐ ChIP-seq
☒	☐ Flow cytometry
☒	☐ MRI-based neuroimaging

Plants

Seed stocks	n/a
Novel plant genotypes	n/a
Authentication	n/a
